# Elective Endovascular Aneurysm Repair (EVAR) for the Treatment of Infrarenal Abdominal Aortic Aneurysms of 5.0–5.5 cm: Differences between Men and Women

**DOI:** 10.3390/jcm12134364

**Published:** 2023-06-28

**Authors:** Ombretta Martinelli, Simone Cuozzo, Francesca Miceli, Roberto Gattuso, Vito D’Andrea, Paolo Sapienza, Maria Irene Bellini

**Affiliations:** 1 Department of General and Speciality Surgery, Sapienza University of Rome, 00161 Rome, Italy; 2 Department of Surgery, Sapienza University of Rome, 00161 Rome, Italy

**Keywords:** abdominal aortic aneurysm, EVAR, sex-related outcomes, surgery

## Abstract

Background: There is significant debate regarding the existence of sex-related differences in the presentation, treatment, and outcomes of men versus women affected by abdominal aortic aneurysm (AAA). The purpose of this study is to compare endovascular aneurysm repair (EVAR) of infrarenal AAAs with the current sex-neutral 5.0–5.5 cm-diameter threshold for intervention between the two sexes. Methods: Retrospective review of consecutive cases from a single teaching institution over a period of five years of patients who had undergone elective EVAR for AAAs between 5.0 and 5.5 cm in diameter. Outcomes of interest were compared according to sex. Results: Ninety-four patients were included in the analysis, with a higher prevalence of men (53%). Females were older at the time of repair, 78 ± 5.1 years, versus 71.7 ± 7 years (*p* < 0.01), and had higher incidence of underlying comorbidities, namely, arrhythmia, chronic kidney disease, and previous carotid revascularization. Women had higher incidence of immediate systemic complications (*p* = 0.021), post-operative AMI (*p* = 0.001), arrhythmia (*p* = 0.006), pulmonary oedema (*p* < 0.001), and persistent renal dysfunction (*p* = 0.029). Multivariate analysis for post-operative factors associated to mortality and adjusted for sex confirmed that AMI (*p* = 0.015), arrhythmia (*p* = 0.049), pulmonary oedema (*p* = 0.015), persistent renal dysfunction (*p* < 0.001), cerebral ischemia (*p* < 0.001), arterial embolism of lower limbs (*p* < 0.001), and deep-vein thrombosis of lower limbs (*p* < 0.001) were associated to higher EVAR-related mortality; a higher incidence of post-operative AMI (*p* = 0.014), pulmonary edema (*p* = 0.034), and arterial embolism of lower limbs (*p* = 0.046) were associated to higher 30-days mortality. In females there was also a higher rate of suprarenal fixation (*p* = 0.026), insertion outside the instruction for use (*p* = 0.035), and a more hostile neck anatomy with different proximal aortic diameter (*p* < 0.001) and angle (*p* = 0.003). Conclusions: A similar threshold of size of AAA for elective surgery for both males and females might not be appropriate for surgical intervention, as females tend to have worse outcomes. Further population-based studies are needed to guide on sex-related differences and intervention on AAA.

## 1. Introduction

Abdominal aortic aneurysm (AAA) is one of the most threatening vascular diseases with a significatively higher prevalence in men [1]. Despite women have lower probability to develop infrarenal AAA, when it occurs, they are four times more likely to experience AAA rupture [2]. The current standard of care is to delay surgery until the AAA is ≥5.5 cm for men and ≥5.0 cm in women [3], because AAA rupture occurs at smaller diameters in women than in men, therefore, the burden associated with AAA is likely to differ because of sex-based characteristics. To use a similar size threshold for elective surgery for both males and females might not be appropriate [4] and the “maximum diameter criterion”, as a single parameter that fits all patients, is not considered as an absolute indication for surgery [5].

Literature suggests that elective surgical repair does not confer a tangible advantage until the diameter of the aneurysm reaches 5–5.5 cm [6], so it could be asserted that there is enough evidence to support a watchful waiting for small AAAs [7] and a prompt repair for larger ones, using the above data as a cut-off value. The female under-representation in surgical research may lead to a significant persistent sex bias [8], without thoroughly assessing the clinical features of the disease [9].

Endovascular aortic aneurysm repair (EVAR), is widely proven to have better early results when compared to open surgery (OS) [10], although there is evidence that females who received either OS or EVAR, remain at higher risk of postoperative mortality than males [11]. Previous studies showed the disparities of risks and outcomes of AAA between the two sexes, on the basis of a unique treatment indistinctly used either electively or in emergency [12,13,14], with far worse outcomes in females. Furthermore, there are inherent morphological dissimilarities between male and female patients, as the disease is intrinsically different among the two sexes [15].

The aim of the present study is to define the sex-related differences of non-ruptured infrarenal AAA of 5.0–5.5 cm in size treated with EVAR at our institution.

## 2. Methods

Retrospective analysis was conducted of an anonymized database of prospectively collected consecutive cases from a single teaching institution over a period of five years. Approval was obtained from the institution’s review board (IRB) of the Department of Surgery and the study was conducted according to the Declaration of Helsinki principles.

### 2.1. Inclusion Criteria

Only patients who underwent elective EVAR for infrarenal AAA with a diameter between 5 and 5.5 cm between January 2017 and January 2022 were included in the study.

### 2.2. Exclusion Criteria

Emergency cases, hybrid procedures, and endovascular AAA repair using iliac branch device or aorto-uni-iliac stent graft, chimney technique, fenestrated graft, and branched stent graft, to overcome challenging proximal landing zones, were excluded. 

### 2.3. Variables Considered

Two cohorts were grouped according to sex: females (group 1) and males (group 2). Comparison was then performed considering demographic information, comorbidities, previous history of thoracic aortic aneurysm, the morphological characteristics of AAAs, type of graft, pre- and operative findings and, in-hospital or EVAR-related, perioperative (30-days mortality from the surgical operation) and long-term survivals (five years). 

Severe aorto-iliac atherosclerotic disease was defined as the concomitant presence of diffuse multiple stenosis (longer than 10 cm in length) involving the aortic bifurcation, the common and external iliac arteries and non-severe atherosclerotic disease as the concomitant presence of multiple stenosis (shorter than 10 cm in length) involving the aortic bifurcation, the common and external iliac arteries, to determine an eventual hamper in EVAR positioning.

### 2.4. Preoperative Work-Up and Surgical Procedure

Preoperative computed tomography (CT) anatomic measurements from the axial, sagittal, and coronal views, and three-dimensional reconstructions, using Osirix Dicom workstation, Pixmeo SARL (CH), were used to identify the aneurysms, their size (in millimeters) and morphology, location, iliac artery involvement, and tortuosity of the iliac–femoral axis. Length measurements of the intended proximal landing zone were measured to choose the main body of the endograft and to assess the adequacy of the proximal aortic neck. The angle of the aortic neck was measured between the aortic neck and the longitudinal axis of the aneurysm seen on maximum-intensity projection images (MIP) with terminal points of measurement at the proximal infrarenal aortic neck and at the aortic bifurcation situated in the center lumen line and the apex of the angle located at the distal point of the infrarenal aortic neck.

Even though procedural steps might have been slightly different according to the stent graft systems, bifurcated endograft were always used and EVAR procedure was performed through percutaneous access or surgical cut-down of both common femoral arteries under local anesthesia. Completion arteriography was always conducted to confirm the patency of all stent graft components and to exclude endoleaks. 

According to operator preferences and familiarity with the different devices, aortic cuff or devices with suprarenal fixation or stent graft with infrarenal fixator were applied taking into consideration patient’s proximal neck anatomy. Additional modular components, stents, or angioplasty were employed to enhance sealing and to overcome challenging iliac landing zones.

The embolization of the aneurysmal sac or of branch arteries emerging from AAAs, such as accessory renal, inferior mesenteric, and lumbar arteries and of the internal iliac arteries, was performed on a selective case based on the diameter of the branching vessels, the true lumen of the aneurysm, and the need of the landing zone in the external iliac. According to the reporting standards for EVAR [15], we considered the following: technical success was the successful delivery and deployment of the aortic stent graft and the aneurysm sac exclusion without complications such as mortality, endoleaks, graft limb obstruction, or surgical conversion occurring during the procedure. Clinical success was reported on an intent-to-treat basis as successful endovascular procedure in absence of death, endoleaks, aneurysm sac expansion greater than diameter 5 mm in six months, aneurysm rupture, graft infection or obstruction, conversion to open surgical repair, and complications (such as permanent paraplegia, disabling stroke, and permanent dialysis). 

### 2.5. Postoperative Course and Surgical Follow-Up

In agreement with the European Society of Vascular Surgery (ESVS) guidelines [3], follow-up protocol included color duplex ultrasound (CDUS) and CT angiography after the procedure, followed by CDUS at 3, 6, and 12 months and, then, annual CDUS control with CT angiography reserved for early seal assessment at 1 month and at 5 years or for confirmatory imaging of one or three endoleaks or sac expansion with or without endoleaks, as detected by CDUS or when CDUS was non-diagnostic.

The hospital course including admission to the intensive-care unit (ICU) and postoperative complications as acute myocardial infarction (AMI), pulmonary oedema and pneumonia, stroke, persistent renal dysfunction, and inferior limbs arterial and venous thrombosis were reviewed. In-hospital mortality was defined as EVAR-related mortality; perioperative mortality within 30 days from the operation, and long-term mortality at five-year follow-up.

### 2.6. Statistical Analysis

SPSS version 27 was used for the analysis. Continuous variables are presented as mean ± standard deviation and compared using one-way ANOVA. Ordinal and dichotomous variables with frequency are compared with chi-squared test and presented with median and ranges. A multivariate analysis of post-operative factors related to mortality and adjusted for sex was run. The Kaplan–Meier method was applied for survival analysis. We took the *p* value as less than 0.05 for statistical significance. 

## 3. Results

### 3.1. Baseline Characteristics

Ninety-four patients were included in the analysis, 50 males (53%), Table 1. Mean age was 74.7 ± 6.9 years for the whole cohort: more in detail 78 ± 5.1 years for females (group 1) and 71.7 ± 7 years for males (group 2) (*p* < 0.001). Significant differences were also noted for the ASA (American Society of Anesthesiologists) score (*p* < 0.015), which was higher for the first group: median 3 (range 1–4) vs. 2 (range 1–3) in group 2. 

In group 1, a higher incidence was present for chronic kidney disease (*p* = 0.028), (25/44, 57%, vs. 11/50, 22%), previous stroke (*p* = 0.01), (6/44, 14% vs. 6/50 12%) and carotid revascularization (*p* < 0.001), (8/44, 18% vs. 4/50, 8%). Group 2 had, instead, a higher incidence of severe atherosclerotic disease of the aorta (*p* = 0.02), (38/50, 76%, vs. 1/44, 2%).

All thoracic aneurysms involved the ascending aorta, except two of the descending aorta, one in each group. The mean diameter of the thoracic aneurysms was 38.6 mm for group 1 and was 39 mm of group 2, therefore, they were not treated. 

As expected, there were also differences regarding the morphological features of the iliac–femoral axes, being larger in group 2: 10 mm mean diameter versus 8 mm for the iliac arteries and 10 mm versus 7 mm for the femoral vessels (*p* < 0.01), Table 2. Significant difference was observed with regards to the proximal aortic diameter and angulation, but not for the aortic neck length. The morphological aspects of the proximal aortic neck are reported in Table 3.

### 3.2. Postoperative Course

EVAR procedure had a higher rate of suprarenal fixation in group 1 (24/44, 54% versus 19/50, 38%) (*p* = 0.026) and was placed more frequently outside the IFU (*p* = 0.035), Table 4. Multivariate analysis for factors related to mortality and adjusted for sex is presented in Table 5. Primary technical success was 100% for both groups. The estimated short-term and mid-term primary clinical success rates were 86.5% and 65.1% for group 1, and 89.8% and 65.5% for group 2. Table 6 summarizes the type of grafts used in both groups.

Endoleak incidence did not differ between the two sexes (25/94, 26%), as well as the rate of endovascular re-intervention (25/94, 27%) and admission to ICU (5/94, 5%). Types of endoleak for each group are reported in Table 7.

The overall incidence of systemic complications occurring after surgery (35/94, 37%) was higher in females. Major complications for the whole cohort were AMI (18/94, 19%), arrhythmia (14/94, 15%), pulmonary oedema (14/94, 15%), respiratory failure (22/94, 23%), persistent renal dysfunction (5/94, 5%), cerebral ischemia (4/94, 4%), arterial embolism of lower limbs (5/94, 5%), and deep venous thrombosis of lower limbs (4/94, 4%). The overall incidence of systemic complications following EVAR was 35/94 cases (37%). There were no statistically significant differences between the EVAR stent graft types, in terms of intraoperative, perioperative, and late complication rates except for Nellix which was burdened by a higher incidence of type 1a endoleaks, surgical migrations, and hospitalizations (10%).

### 3.3. Mortality

Mean follow-up was 36.8 ± 23.2 months (range, 14–62 months). Mortality did not statistically differ for the two groups, as shown below in Table 8.

The systemic causes of early (30 days) and late (5 years) mortality consisted in: AMI in seven cases (11.3% in women and 4% in men), arrhythmia in two women, acute renal failure in one woman, pneumonia in one man, and pulmonary embolism in one man. In the multivariate analysis, female perioperative mortality was associated to the following factors: AMI (0.014), pulmonary oedema (0.034), and arterial embolism of lower limb (0.046); as noted previously, group 1 had higher incidence of AMI and pulmonary oedema. The same consideration applies for EVAR-related mortality, with the factors being associated consisting of: AMI (*p* = 0.015), arrhythmia (*p* = 0.049), pulmonary oedema (*p* = 0.015), persistent renal dysfunction (*p* < 0.001), cerebral ischemia (*p* < 0.001), arterial embolism of lower limbs (*p* < 0.001), and deep venous thrombosis of lower limbs (*p* < 0.001). 

In the group 1, EVAR-related (in-hospital) mortality was due to: type I A endoleak and graft migration with subsequent AAA rupture in two cases, perioperative IMA following secondary intervention for type 1A endoleak in one case and acute respiratory failure following endograft explantation. In Group 2, the causes of EVAR-related mortality were: perioperative IMA and pneumonia in two cases of graft removal and one acute kidney failure after secondary procedure to correct combined type 1 A and type II endoleaks.

## 4. Discussion

Abdominal aortic aneurysm is significantly more common in the male population with a three- to four-times higher rate, therefore, women were usually under-represented in the main clinical trials which established the current guidelines and threshold for treatment [3]. Yet, although AAAs occur less frequently in females, in this setting, they have a higher rupture risk, occurring at smaller diameters. This may be explained by the fact that women have smaller native aorta, thus, aneurysms of the same size as men represent a more advanced disease stage. Additionally, women have a major inflammatory component of the aortic aneurysmal wall, which may lead to faster growth and rupture than in men even for small AAAs [16,17]. 

Currently, EVAR has been widely accepted as the primary treatment for elective AAA repair due to its minimal invasiveness and lower early mortality and morbidity than conventional surgery, even if its durability over time is still questionable [18]. 

From our data, the early advantages of EVAR are less significant when the outcomes are analyzed according to sex: despite the same EVAR treatment within the same institution, our study showed women had apparent higher rates of deaths from all causes than men, both during the procedure hospitalization and during the first 30 days after discharge, although this result did not reach statistical significance. This trend is confirmed by a systematic review, reporting that operative mortality for elective EVAR in women is 2.9% vs. 1.5% in men [19].

Importantly, we detected a higher incidence of post-operative systemic complications (Table 4), namely, post-operative AMI, arrhythmia, pulmonary oedema, and persistent renal dysfunction in group 1, that at the multivariate analysis for factors associated with mortality and adjusted for sex, confirmed what literature reports for higher female risk: limb ischemia, renal complications, and cardiac complications [20].

Given that the intervention itself is a major risk factor, the adverse outcomes of female patients may also be explained by their older age, which is often associated with more severe comorbidities and greater frailty. 

In our cohort, compared with males, women were older and had a higher incidence of chronic kidney disease (*p* = 0.02), (25/44, 57%, vs. 11/50, 22%), which might also justify the higher ASA score in females. Significantly, there was a higher rate of female patients with previous stroke (*p* = 0.01) (6/44, 14% vs. 6/50 12%) and carotid artery disease that required previous revascularization (*p* < 0.001) (8/44, 18% vs. 4/50, 4%), too. Based on the concept that atherosclerosis affects both carotid and coronary systems, although not always in identical phenotypic manner, previous carotid disease may predict the presence of severe coronary disease and cardiovascular complications and death after EVAR [21,22]. Additionally, renal insufficiency and arrhythmia were also more present preoperatively in group 1 (Table 1), in agreement with the labeling of this cohort as at “higher risk”.

Earlier studies demonstrated the same results but a comprehensive evidence-based study is still lacking [23,24]. Furthermore, to our knowledge, none of them have compared the outcomes after elective EVAR for intact AAA with sizes at the lower limits of the cut-off for treatment.

In the present study, the worse outcomes of women seem to be primarily driven by preexisting comorbidities, therefore, an optimal preoperative medical management can be particularly crucial for women to reduce this higher risk following elective EVAR. Additionally, if the starting point of the native aorta is smaller for women, the same size threshold to treat AAAs could not be considered; instead, there is a need to focus on improving AAA outcomes in females, preferably with a repair at a smaller size cut-off. Thus, a tailored AAA management conformed to their specific characteristic. Notably, an earlier stage of intervention could be linked with fewer comorbidities, as, in general, they tend to progress with age and with a synergistic interaction.

It is well accepted that the basic requirement for EVAR feasibility relies on a suitable morphology of AAA and of the proximal aorta and iliac arteries for easy introduction of the device and proper endograft anchoring. It follows that a hostile proximal aortic neck and unfavorable iliac anatomy are potential contributing factors for worse outcomes of EVAR [25]. Given the above, in our series, women undergoing EVAR had different proximal aortic neck morphology and iliac anatomy, despite the same AAA size of men (Table 3).

Consequently, in group 1, there was a higher rate of suprarenal fixation to minimize the risk of migration due to a more hostile infrarenal neck anatomy. Though there is no definite disadvantage to pararenal bare-metal stents and accompanying suprarenal fixation, the suprarenal implantation of EVAR devices, especially with hooks or barbs, may interfere with blood flow to the renal arteries [26]. Additionally, if it ever becomes necessary, the endograft removal is more complicated and requires suprarenal clamping.

One third of female patients did not meet device IFU mainly due to larger and more angulated, but not shorter, proximal necks and inadequate access vessel size. The more advanced age and the suboptimal surgical risk profile influenced the decision to perform EVAR outside-IFU in this group. Albeit a more challenging anatomy for EVAR and the largest number of off-label procedures carried out in the female group, the rates of all types of endoleak, especially those requiring reintervention were similar between the two sexes, suggesting that the operators’ experience in EVAR surgical planning and the extension of the effective seal length beyond the anatomic neck may influence the outcomes, despite IFU nonadherence [27]. Group 1 also experienced long-term survival, aneurysm-free survival, and rates of reintervention equivalent to those of their male counterpart.

Consistently with a growing body of literature [28,29], our results of off-label EVAR seem to match the results of on-label procedures, suggesting that IFUs are not inviolable, but the surgeon’s judgment at the moment of the intervention, guided by his/her own experience and the overall morphological characteristics of the patient, as well as the underlying comorbidities, is the final decision-maker to take responsibility of an eventual IFU violation and to what extent. It is worth mentioning that EVAR is fragile to late aortic changes, especially in hostile aortic neck anatomy, with a propensity toward reinterventions and related complications, so IFUs represent mostly a legal value as they protect the industry and the surgeon who uses the prosthesis inside the IFU, in case of complications. This may reinforce the importance of performing off-label EVAR as a last-resort strategy. 

Hostile proximal aortic neck is also known to be associated with increased risk of perioperative mortality after EVAR [30]; this further confirms the importance of tailor-made devices for these high-risk patients. In this regard, modern endografts, with lower profile, widely applicable IFU, and greater conformability, are better suited for the complex anatomy often seen in women. Particularly, the use of low-profile devices and techniques such as endoanchors may overcome anatomical restrictions, especially those encountered in females [31,32].

The main limitation of this study is its retrospective nature. Furthermore, we only analyzed the data of patients undergoing elective EVAR to treat AAA of 5.0–5.5 cm; inherently, the number of cases was small and the follow-up limited. Finally, although the reported analyses included many relevant clinical and operative details, it is possible that unmeasured factors contribute to different outcomes of EVAR between the two sexes.

Our findings remark that the current diameter thresholds alone are not accurate enough indicators for AAA elective repair because several challenges, including sex, patient’s age and risk factors, can influence the outcomes and the decision making [33].

When evaluating a patient with AAA, it is necessary to explore the best therapeutic approach based on the risk of rupture and the overall benefit of the intervention. Since women tend generally to experience poorer outcomes, interventions aiming towards amelioration of gender-related medical treatment should be welcome and encouraged. The continuous evolution of EVAR will further improve the outcomes of treatment of both elective and urgent AAA repair in both sexes.

In conclusion, this study suggests that females with AAAs at a diameter 5.0–5.5 cm usually present at an older age and with a higher risk than males. More insight into gender-related clinical and anatomic differences and an increased awareness of the inferior outcomes in women may influence the treatment strategies leading to sex-specific management and new guidelines with lower cut-off size of AAA to improve results in this cohort of patients.

## Figures and Tables

**Table 1:** Preoperative group characteristics. Brackets report the relative percentage.

Variable	Group 1 (Females)	Group 2 (Males)	*p*
Preoperative AMI	11 (25)	17 (34)	ns
Arrhythmia	12 (50)	22 (24)	<0.001
Arterial hypertension	36 (81)	38 (76)	ns
COPD	25 (57)	32 (64)	ns
Chronic kidney disease	25 (57)	11 (22)	0.028
Carotid disease	31 (71)	26 (52)	ns
Previous stroke	6 (14)	6 (12)	0.01
Previous carotid revascularization	8 (18)	4 (8)	<0.001
Severe atherosclerotic disease of the aorta	1 (2)	38 (76)	0.02
Thoracic aortic aneurysm	6 (14)	6 (12)	ns
Chronic peripheral arterial disease	4 (9)	3 (6)	ns
Previous deep venous thrombosis	10 (23)	6 (12)	ns

AMI: acute myocardial infarction; COPD: chronic obstructive pulmonary disease.

**Table 2:** Iliac–femoral axis dimensions in the cohort.

Variable (mm)	Group 1 (Females)	Group 2 (Males)	*p*
AAA Axial diameter: mean ± st. dev. (range)	52.68 ± 2.055 (49–56)	53.18 ± 2.195 (49–57)	0.722
Diameter right iliac axis	7.85 ± 0.838 (6–10)	10.04 ± 2.98 (8–13)	<0.01
Diameter left iliac axis	8.1 ± 0.907 (6–10)	10.96 ± 2.02 (8–16)	<0.01
Diameter right femoral axis	7.26 ± 0.741 (6–8)	9.66 ± 1.2 (7–12)	<0.01
Diameter left femoral axis	7.1 ± 0.74 (6–8)	9.64 ± 1.02 (7–12)	<0.01
Neck length	15.8 ± 4.3 (7–24)	17.7 ± 5.3 (7–28)	<0.01
Neck diameter	25.8 ± 1.9 (21–30)	22 ± 3 (16–28)	<0.01
Neck angle	49 ± 9.8 (27–68)	40.1 ± 10 (23–64)	<0.01

AAA: abdominal aortic aneurysm.

**Table 3:** Morphological characteristics of proximal aortic neck. Brackets report the relative percentage.

Variable	Group 1 (Females)	Group 2 (Males)	*p*
Length (mm)			*p* = 0.56
≥15	21 (47.7)	35 (70.0)	
≥10 length <15	21 (47.7)	13 (26.0)	
≤10	2 (4.5)	2 (4)	
Total	44 (100)	50 (100)	
Diameter (mm)			*p <* 0.001
<24	3 (6.8)	35 (70.0)	
24 ≤ diameter ≤ 26	20 (45.5)	12 (24.0)	
>26	21 (47.7)	3 (6.0)	
Total	44 (100)	50 (100)	
Angle (°)			*p* = 0.003
<40°	10 (22.7)	28 (56.0)	
40 ≤ angle ≤ 60	25 (56.8)	18 (36.0)	
>60	9 (20.5)	4 (8)	
Total	44 (100)	50 (100)	

**Table 4:** Postoperative group characteristics. Brackets report the relative percentage.

Variable	Total	Group 1 (Females)	Group 2 (Males)	*p*
EVAR suprarenal fixation	43 (46)	24 (55)	19 (38)	0.026
EVAR inserted outside instruction for use *	26 (28)	16 (36)	10 (20)	0.035
Endoleak	35 (37)	17 (39)	18 (36)	ns
Vascular reintervention	25 (27)	11 (25)	14 (28)	ns
ICU admission	81 (86)	37 (84)	44 (88)	ns
Immediate systemic complications	35 (37)	21 (48)	14 (28)	0.021
Post-operative AMI	18 (19)	14 (32)	4 (8)	0.001
Arrhythmia	17 (18)	12 (27)	5 (10)	0.006
Pulmonary oedema	14 (15)	12 (27)	2 (4)	<0.001
Pneumonia	22 (23)	11 (25)	11 (22)	ns
Persistent renal dysfunction	5 (5)	4 (9)	1 (2)	0.029
Cerebral ischemia	4 (4)	3 (7)	1 (2)	ns
Arterial embolism of lower limbs	5 (5)	2 (5)	3 (6)	ns
Deep venous thrombosis of lower limbs	4 (4)	3 (7)	1 (2)	ns
EVAR-related (in-hospital) mortality	7 (7)	4 (9)	3 (6)	ns
Perioperative (30 days) mortality	8 (9)	6 (14)	2 (4)	ns
Long-term (5 years) mortality	5 (5)	2 (5)	3 (6)	ns

* Standard IFU was defined as follows: proximal aneurysm neck diameter 18–32 mm, neck angulation < 60 degrees, infrarenal neck length > 10 mm, iliac diameter 8–22 mm, and distal fixation length > 15 mm were considered to meet instruction for use (IFU) adherence of EVAR—off-label EVAR was performed exclusively in cases involving challenging neck or AAA anatomy. EVAR: endovascul araneurysm repair.

**Table 5:** Multivariate analysis of factors related to early and EVAR-related mortality and adjusted for sex.

Factor	Perioperative Mortality (*p*)	EVAR-Related Mortality (*p*)
Immediate systemic complications	0.318	0.607
AMI	0.014	0.015
Arrythmia	0.248	0.049
Pulmonary oedema	0.034	0.015
Pneumonia	0.093	0.263
Persistent renal dysfunction	0.119	<0.001
Cerebral ischemia	0.081	<0.001
Arterial embolism of lower limb	0.046	<0.001
Deep venous thrombosis of lower limbs	0.119	<0.001

AMI: acute myocardial infarction; EVAR: endovascul araneurysm repair.

**Table 6:** Type of graft used during surgery.

Type of Graft	Total	Group 1 (Females)	Group 2 (Males)	*p*
Zenith endovascular grafts (Cook Medical, Bloomington, IN, USA)	11	6	5	ns
Gore Excluder AAA Endoprosthesis (W.L. Gore & Associates, Inc., Flagstaff, AZ, USA)	19	9	10	
Nellix (Endologix, Irvine, CA, USA)	22	7	15	
AFX endografts (Endologix Inc., Irvine, CA, USA)	22	14	8	
Ovation stent graft (Endologix, Irvine, CA, USA)	4	2	2	
Treovance stent grafts (Bolton Medical, Barcelona, Spain)	2	2	0	
Incraft aortic stent grafts (Cordis, Bridgewater, NJ, USA)	5	3	2	
Endurant stent graft (Medtronic, Minneapolis, MN, USA)	9	1	8	
Total	94	44	50	

AAA: abdominal aortic aneurysm.

**Table 7:** Post-EVAR endoleak incidence. Brackets report the relative percentage.

	Women	Men	*p*
No	32 (73)	42 (7)	ns
IA	4 (9)	5 (9)	
IB	1 (2)	2 (4)	
II	7 (16) of which 5 (14) spontaneously resolved	5 (9) of which 3 (5) spontaneously resolved	
III	0	1 (2)	

**Table 8:** (**a**). In-hospital mortality probability. Log-rank 0.144. (**b**). 30 days mortality probability. Log-rank 0.731. (**c**). Long-term mortality probability. Log-rank 0.332.

(a)				
In-Hospital Mortality	Mean			
	Estimate	Std. Error	95% Confidence Interval	
			Lower Bound	Upper Bound
Females	53.291	3.187	47.045	59.537
Males	56.800	1.753	53.365	60.235
Overall	55.913	1.876	52.237	59.590
(**b**)				
**30-Days Mortality**	**Mean**			
	**Estimate**	**Std. Error**	**95% Confidence Interval**	
			**Lower Bound**	**Upper Bound**
Females	55.851	2.862	50.242	61.460
Males	56.091	2.092	51.992	60.190
Overall	57.249	1.695	53.927	60.572
(**c**)				
**Long-Term Mortality**	**Mean**			
	**Estimate**	**Std. Error**	**95% Confidence Interval**	
			**Lower Bound**	**Upper Bound**
Females	59.636	1.478	56.740	62.532
Males	56.629	1.843	53.017	60.241
Overall	58.573	1.416	55.798	61.347

## Data Availability

Raw data were generated at Sapienza University. Derived data supporting the findings of this study are available from the corresponding author on request.

## Abbreviations

AAA	abdominal aortic aneurysm
ASA	American Society of Anesthesiologists
COPD	chronic obstructive pulmonary disease
CDUS	color duplex ultrasound
CT	computed tomography
IMA	acute myocardial infarction
EVAR	endovascular aneurysm repair
MIP	Maximum-intensity projection images
OS	open surgery
